# Evidence for increased breakthrough rates of SARS-CoV-2 variants of concern in BNT162b2-mRNA-vaccinated individuals

**DOI:** 10.1038/s41591-021-01413-7

**Published:** 2021-06-14

**Authors:** Talia Kustin, Noam Harel, Uriah Finkel, Shay Perchik, Sheri Harari, Maayan Tahor, Itamar Caspi, Rachel Levy, Michael Leshchinsky, Shifra Ken Dror, Galit Bergerzon, Hala Gadban, Faten Gadban, Eti Eliassian, Orit Shimron, Loulou Saleh, Haim Ben-Zvi, Elena Keren Taraday, Doron Amichay, Anat Ben-Dor, Dana Sagas, Merav Strauss, Yonat Shemer Avni, Amit Huppert, Eldad Kepten, Ran D. Balicer, Doron Netzer, Shay Ben-Shachar, Adi Stern

**Affiliations:** 1 The Shmunis School of Biomedicine and Cancer Research, George S. Wise Faculty of Life Sciences, Tel Aviv University, Tel Aviv, Israel; 2 Edmond J. Safra Center for Bioinformatics, Tel Aviv University, Tel Aviv, Israel; 3 Clalit Research Institute, Innovation Division, Clalit Health Services, Ramat Gan, Israel; 4 Clalit Health Services, Central Laboratories, Haifa and Western Galilee, Nesher, Israel; 5 Progenin Laboratories, Jerusalem District, Clalit Health Services, Tel Aviv, Israel; 6 Microbiology lab, Rabin Medical Center, Beilinson Hospital, Petah Tiqva, Israel; 7 Central Laboratory, Clalit Health Services, Tel Aviv, Israel; 8 Department of Clinical Biochemistry and Pharmacology, Faculty of Health Sciences, Ben Gurion University of the Negev, Beersheba, Israel; 9 Microbiology Laboratory, Emek Medical Center, Afula, Israel; 10 Laboratory of Clinical Virology, Soroka University Medical Center, Beersheba, Israel; 11 Faculty of Health Sciences, Ben Gurion University of the Negev, Beersheba, Israel; 12 The Bio-statistical and Bio-mathematical Unit, The Gertner Institute for Epidemiology and Health Policy Research, Chaim Sheba Medical Center, Tel HaShomer, Ramat Gan, Israel; 13 The Sackler Faculty of Medicine, Tel-Aviv University, Tel Aviv, Israel; 14 Clalit Health Services, Tel Aviv, Israel

**Keywords:** SARS-CoV-2, Phylogenetics, Viral infection

## Abstract

The BNT162b2 mRNA vaccine is highly effective against SARS-CoV-2. However, apprehension exists that variants of concern (VOCs) may evade vaccine protection, due to evidence of reduced neutralization of the VOCs B.1.1.7 and B.1.351 by vaccine sera in laboratory assays. We performed a matched cohort study to examine the distribution of VOCs in infections of BNT162b2 mRNA vaccinees from Clalit Health Services (Israel) using viral genomic sequencing, and hypothesized that if vaccine effectiveness against a VOC is reduced, its proportion among breakthrough cases would be higher than in unvaccinated controls. Analyzing 813 viral genome sequences from nasopharyngeal swabs, we showed that vaccinees who tested positive at least 7 days after the second dose were disproportionally infected with B.1.351, compared with controls. Those who tested positive between 2 weeks after the first dose and 6 days after the second dose were disproportionally infected by B.1.1.7. These findings suggest reduced vaccine effectiveness against both VOCs within particular time windows. Our results emphasize the importance of rigorously tracking viral variants, and of increasing vaccination to prevent the spread of VOCs.

## Abstract (web summary)

At early time points after vaccination with a single dose or two doses of the BNT162b2 mRNA COVID-19 vaccine, breakthrough SARS-CoV-2 infections can be disproportionately caused by the B.1.1.7 or B.1.351 variants of concern, underlining the need to ensure rapid and complete vaccination.

## Main

Mass vaccination against severe acute respiratory syndrome coronavirus 2 (SARS-CoV-2) is currently underway worldwide, providing hope that the coronavirus disease (2019) COVID-19 pandemic may soon be mitigated. In Israel, vaccination commenced on 20 December 2020, primarily with the BNT162b2 mRNA vaccine, and by mid-March 2021, more than 80% of the eligible population (all individuals 16 years old and above) were vaccinated with at least one dose. In clinical trials, the BNT162b2 mRNA vaccine was shown to be 95% efficacious in preventing symptomatic disease; a similarly high protective effectiveness has also been found in real-world settings in Israel^1,2^. However, concerns have emerged regarding the effectiveness of vaccines against various SARS-CoV-2 strains. In particular, three strains have recently been defined as VOCs by the WHO (World Health Organization): the B.1.1.7 strain (first detected in the UK), the B.1.351 strain (first detected in South Africa) and the P.1 strain (first detected in Brazil). Accumulating evidence suggests that the B.1.1.7 strain spreads more rapidly than the original circulating strain and leads to substantially more infections^3,4^.

Concerns have emerged that the B.1.351 and P.1 strains are able to overcome previous immunity to SARS-CoV-2 (refs. ^5,6^), yet the evidence has been mixed. Using engineered viruses and/or sequences, laboratory studies have shown that neutralization of B.1.1.7 by BNT162b2-vaccine-elicited sera was either similar to or slightly reduced as compared to neutralization of early circulating isolates^7–12^ with or without the globally dominant D614G alteration^13^. Conversely, a significant reduction in neutralization of B.1.351 was observed^7–12^, while other studies suggested neutralization remained relatively high against both B.1.1.7 and B.1.351 (ref. ^14^). T cell responses, which are not captured by neutralization studies, were also shown to remain stable against these variants following vaccination^15^. Thus, it remains unknown whether VOCs can mediate BNT162b2 vaccine breakthrough in real-world settings, in which the vaccine elicits persistent antibody and T cell responses. Here, we tested the hypothesis that the B.1.1.7 and B.1.351 strains are able to overcome BNT162b2 mRNA vaccine protection, by comparing their distributions in infected vaccinated individuals and in infected non-vaccinated individuals.

## Results

### Study population

We began by identifying the relatively rare vaccinees with documented SARS-CoV-2 infection—symptomatic or asymptomatic—among members of Clalit Health Services (CHS), the largest health care organization in Israel, which insures 4.7 million patients (53% of the population). We divided these individuals into two categories: individuals who had a positive PCR test that was performed between 14 days after the first dose and 6 days after the second dose (denoted as the dose1 group); and individuals who had a positive PCR test that was performed at least 7 days after the second vaccine dose (denoted as the dose2 group). The definitions of these two categories were chosen to match the original BNT162b2 vaccine efficacy study^2^, as well as our ensuing real-world effectiveness study in Israel^1^, both of which revealed very high vaccine protection using these particular criteria. Each vaccinee (case) was matched with an unvaccinated infected individual (control) who tested positive on a similar date (±3 days) and had similar demographic characteristics (age, sex, ethnic sector and geographic location) to reduce bias associated with differential exposure (Methods). Next, we obtained RNA from the nasopharyngeal swabs sampled for PCR and performed complete viral genome sequencing for 813 samples from different individuals, consisting of 149 pairs of dose2–controls, 247 pairs of dose1–controls and additional samples whose match did not undergo successful sequencing (see below; Table 1, Supplementary Table 1 and Supplementary Fig. 1).

**Table 1:** Demographic statistics on paired cases and controls sequenced herein

	Control dose2 vaccinee (*n* = 149)	dose2 vaccinee (*n* = 149)	Control dose1 vaccinee (*n* = 247)	dose1 vaccinee (*n* = 247)
Age group				
0–19	2 (1.3)		4 (1.6)	1 (0.4)
20–29	20 (13.4)	5 (3.4)	31 (12.6)	31 (12.6)
30–39	32 (21.5)	12 (8.1)	59 (23.9)	48 (19.4)
40–49	33 (22.1)	31 (20.8)	59 (23.9)	64 (25.9)
50–59	22 (14.8)	24 (16.1)	55 (22.3)	53 (21.5)
60–69	24 (16.1)	30 (20.1)	25 (10.1)	32 (13.0)
70–79	9 (6.0)	24 (16.1)	10 (4.0)	13 (5.3)
80–89	7 (4.7)	22 (14.8)	4 (1.6)	5 (2.0)
90+		1 (0.7)		
Sex				
Female	87 (58.4)	81 (54.4)	152 (61.5)	152 (61.5)
Male	62 (41.6)	68 (45.6)	95 (38.5)	95 (38.5)
District				
Dan	25 (16.8)	23 (15.4)	22 (8.9)	22 (8.9)
South	3 (2.0)	3 (2.0)	5 (2.0)	5 (2.0)
Haifa	45 (30.2)	45 (30.2)	70 (28.3)	70 (28.3)
Jerusalem	29 (19.5)	29 (19.5)	71 (28.7)	71 (28.7)
Center	23 (15.4)	23 (15.4)	28 (11.3)	28 (11.3)
North	7 (4.7)	7 (4.7)	22 (8.9)	22 (8.9)
Sharon-Shomron	7 (4.7)	9 (6.0)	19 (7.7)	19 (7.7)
Tel Aviv	10 (6.7)	10 (6.7)	10 (4.0)	10 (4.0)
Sector				
General Jewish	117 (78.5)	117 (78.5)	163 (66.0)	163 (66.0)
Jewish Orthodox	11 (7.4)	11 (7.4)	28 (11.3)	28 (11.3)
Non-Jewish	21 (14.1)	21 (14.1)	56 (22.7)	56 (22.7)
Vaccine status				
Non-vaccinated	149 (100.0)		247 (100.0)	
14–20 days from first dose				133 (53.8)
21–28 days from first dose				95 (38.5)
28+ days from first dose				19 (7.7)
7–13 days from second dose		73 (49.0)		
14–20 days from second dose		30 (20.1)		
21+ days from second dose		46 (30.9)		

Absolute counts are shown; relative proportions are in brackets.

### Analysis of variant distributions

We next used a stringent method of lineage assignment for each viral sequence (Methods). Aside from B.1.1.7 and B.1.351, no other VOCs or variants of interest, as defined by the WHO, were found in our sample (Supplementary Fig. 2). We hence collectively denoted all non-B.1.1.7 and non-B.1.351 lineages found as wild type (WT). All of these WT lineages bore the D614G alteration, in line with the very high frequency of this alteration across the globe^13^. We did not find evidence for the increased presence of any additional alterations that are not lineage-defining alterations of B.1.1.7 or B.1.351.

**Fig. 1:**
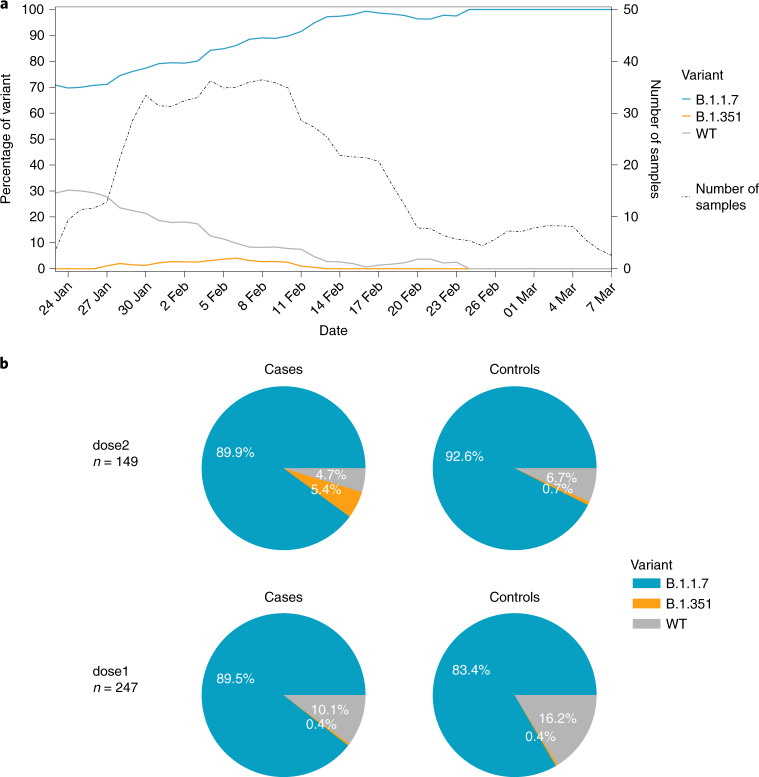
Variant frequencies of SARS-CoV-2-positive samples. **a**, Variant frequencies across the time of the study, including the number of samples collected throughout the study. All values were calculated by averaging over a sliding window of 7 days. All samples sequenced in this study are included herein, including unpaired samples. **b**, Breakdown of variant frequencies based on the four groups of this study. The pie charts display the proportion of each variant (B.1.1.7, B.1.351 and WT) for paired vaccinated cases versus non-vaccinated controls separated by dosage (as defined in the main text), with cases on the left and their associated control on the right. Only paired samples are shown in the figure.

When examining lineage frequency across time, we noted that B.1.1.7 was the predominant strain of the virus in Israel over the entire sampling period (712/813 sequences), increasing in frequency over time (Fig. 1a). Conversely, B.1.351 was at an overall frequency of 1.6% in our sample of both vaccinated and non-vaccinated individuals (13/813 sequences) (Fig. 1b), similar to previous reports of B.1.351 frequency in Israel from January 2021^16^.

**Fig. 2:**
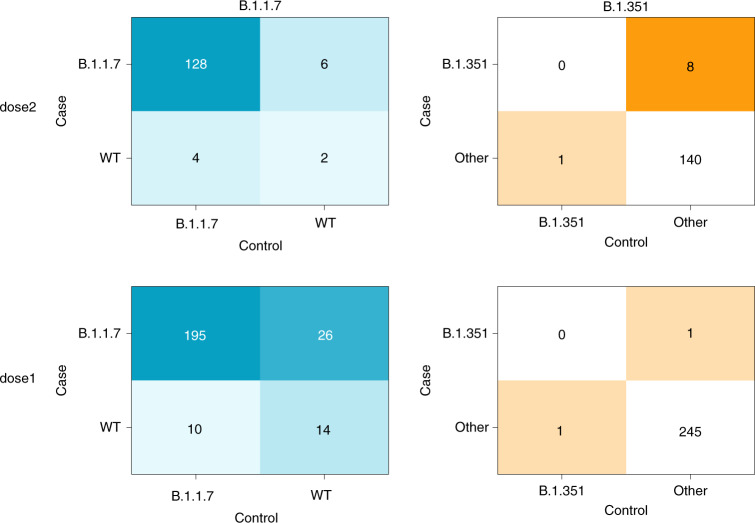
Results of matched vaccinated cases and non-vaccinated controls separated by effectiveness and VOC. In each table, a cell reflects the number of pairs concordant (upper left and lower right) or discordant (upper right or lower left) for a given variant. The left panel focuses on the comparison between B.1.1.7 and WT (pairs with B.1.351 were removed), whereas the right panel focuses on comparing B.1.351 and either WT or B.1.1.7 (denoted collectively as ‘other’). Of note, the McNemar test focuses on a comparison of only discordant samples. Under a null hypothesis of equal vaccine effectiveness against all variants, we expect an equal number of discordant pairs in the upper right cell and the lower left cell, in each of the tables.

On the basis of previous results from neutralization assays, we hypothesized that B.1.1.7 may be slightly vaccine resistant as compared to WT, whereas B.1.351 may be more vaccine resistant when compared to both B.1.1.7 and WT. Under this hypothesis of ordered resistance, we performed our statistical analyses first on the B.1.1.7 strain, while excluding B.1.351 sequences (to avoid obscuring a potential signal), and then compared the B.1.351 with the B.1.1.7 and WT sequences combined (Fig. 2). We use the McNemar test on our paired vaccinees–controls to examine discordant pairs, defined as pairs where a different variant was found in the vaccinee as compared to its matched control. The McNemar test is particularly useful for comparing paired proportions in retrospective cohorts, where each case is paired with a control, as in the study herein. The null model of this test was that under a hypothesis of equal effectiveness of the vaccine against all variants, the different variants should be evenly distributed across the discordant pairs (see Fig. 2 for a more elaborate explanation).

**Fig. 3:**
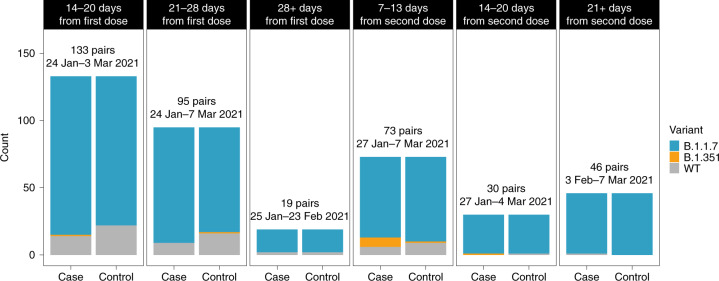
Breakdown of SARS-CoV-2 variant distribution during windows of weeks post vaccination. The first three panels correspond to the dose1 group and the last three panels correspond to the dose2 group. The number of pairs and the isolation date range of the samples are noted for each panel. The dose2 B.1.351 case that is shown in the 14–20 days category was isolated exactly 14 days after the second dose.

No statistically significant difference was observed in the discordant rates of B.1.1.7 infection in dose2 cases versus unvaccinated controls (McNemar odds ratio (OR) of 6:4; one-sided exact McNemar test, *P* = 0.38), but a significantly higher proportion of B.1.351 was observed in dose2 cases versus unvaccinated controls (McNemar OR of 8:1; one-sided exact McNemar test, *P* = 0.02). Of note, about half of dose2 cases tested positive on days 7–13 after the second dose, and about half tested positive 14 days or more after the second dose (Table 1). However, seven out of eight B.1.351 dose2 cases were isolated 7–13 days after the second dose and the eighth B.1.351 dose2 case was isolated exactly 14 days after the second dose (Fig. 3).

On the other hand, a significantly higher rate of B.1.1.7 was observed in dose1 cases versus unvaccinated controls (McNemar OR of 26:10; one-sided exact McNemar test, *P* = 0.006). For B.1.351 in the dose1 category, the sparsity of data (one infection in each category) precluded statistical analysis (Fig. 2). A conditional logistic regression was further performed on the dose1 B.1.1.7 data (as more data were available in this category), supporting the previous analysis: an OR of 2.4 was observed (95% confidence interval of 1.2 to 5.1). Age was included in the regression and was found to be a nonsignificant confounder, suggesting that its possible role in propensity for infection by a specific VOC was corrected through our matching scheme.

### Testing for biases and inclusion of missing data

**Fig. 4:**
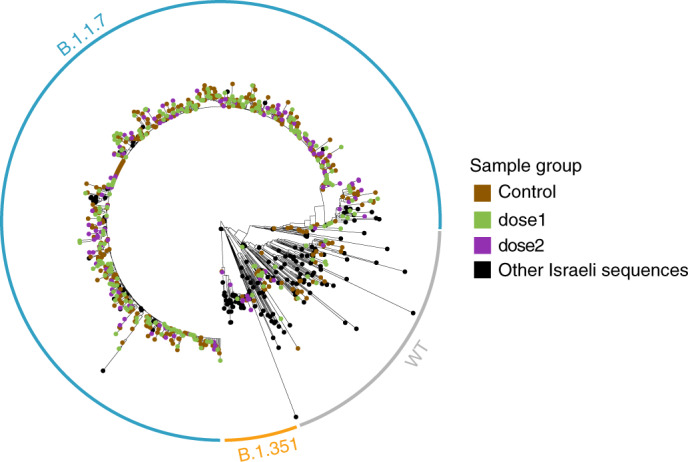
A maximum-likelihood phylogenetic tree of Israeli SARS-CoV-2 samples including those sequenced herein. Vaccinees are colored in violet or green, non-vaccinees are colored in brown, and black sequences are publicly available sequences from Israel (marked as ‘other’, Supplementary Table 2). Clades composed of the B.1.1.7, B.1.351 and WT sequences are encircled in blue, orange and gray, respectively.

To test whether our sampling scheme was biased, we reconstructed a phylogenetic tree of all the sequenced samples together with additional available sequences from Israel, and observed that vaccinated and unvaccinated samples were highly interspersed along the tree (Fig. 4), ruling out strong biases in sampling. Moreover, we focused on the nine dose2 B.1.351 samples (eight cases and one control), and noted that they were from seven different municipalities spread across the geography of Israel. When examining the phylogenetic structure of B.1.351 sequences in Israel in general, we noted that most sequences belonged to one clade whose isolation dates ranged from 28 December 2020 until 9 February 2021, and accordingly most sequences in this clade were quite similar (Supplementary Fig. 5A). Nevertheless, most pairs of dose2 sequences were separated by one to six alterations (Supplementary Fig. 5B). Combined with the data on different municipalities, we conclude that it is unlikely (but not impossible) that these dose2 B.1.351 sequences were part of the same direct transmission chain.

Finally, we noted an additional two B.1.351 sequences, consisting of one dose2 case and one dose1 control, where the sequencing of the matched pair did not undergo successful sequencing, most often due to a high cycle threshold (Ct) value (low viral load (VL)). An additional dose1 control sequence was also ambiguously classified (Methods, Supplementary Fig. 3). Importantly, these sequences would either leave our conclusions regarding B.1.351 unchanged, or would increase the McNemar OR in favor of B.1.351 in the dose2 category (Supplementary Fig. 3), strengthening the results reported above. With regards to B.1.1.7, we found a total of 28 non-paired sequences, once again because a control or case yielded unreliable sequencing. These sequences might change the significance of our results with regards to B.1.1.7 but would not change the trend we found for this variant (Supplementary Fig. 3).

## Discussion

Our results show that there is an increased proportion of VOCs in vaccine breakthrough infections that occurs within two particular windows of time. An increased proportion of B.1.351 was found in individuals fully vaccinated with BNT162b2, 7–14 days after the second dose, as compared to the matched unvaccinated controls. Furthermore, an increased proportion of B.1.1.7 was found in partially vaccinated individuals, 14 days after the first dose until 6 days after the second dose, as compared to the matched unvaccinated control, yet we find no evidence for increased breakthrough rates of B.1.1.7 a week or more after the second dose (Figs. 2 and 3). Not enough data were available to assess vaccine breakthrough of B.1.351 in the dose1 category. These results are generally aligned with those from in vitro neutralization assays that have shown a large reduction in neutralization against B.1.351, and little to no reduction against B.1.1.7 in fully vaccinated individuals^7–11,17^. Overall, our data also suggest that serum-based neutralization studies may provide a good proxy for real-life protection in the case of SARS-CoV-2 (ref. ^18^). Although this remains to be tested in a more widespread manner, it suggests that neutralization studies may be valid as a prompt first step before the establishment of real-world studies in the case of the emergence of new SARS-CoV-2 VOCs.

The power of our approach stems from the combination of real-world evaluation with the stringent case–control matching strategy employed, allowing us to rule out that a high proportion of a given variant was due to a confounding effect. For example, an outbreak of B.1.351 in a given city would have led to spurious identification of infected vaccinees there, yet by matching them with unvaccinated individuals, this confounding effect is controlled for. However, it is still possible that other confounding effects were present and were not controlled for, such as various behavioral effects among vaccinees. Additionally, sequencing limitations prevented us from sequencing very low-VL samples (Methods), and thus the focus of our study was on vaccinees who generated higher VLs. However, it has been shown that cases with a low VL may be a lesser concern from a public health perspective, as they are associated with fewer symptoms and lower risk of transmission^19^. Finally, our dose2 cohort is based on infections documented 7 or more days after the second vaccine dose (Table 1). Some individuals in this cohort may have been infected before the immunity from the boost was fully established, and it is thus possible that enhanced immunity from the boost, which develops over time^20^, may more effectively prevent infection with the B.1.351 variant. Notably, when focusing on the eight B.1.351 cases in the dose2 group, all tested positive during days 7–14 after the second dose, and none tested positive more than 14 days after the second dose. This observation suggests that increased breakthrough of B.1.351 in our cohort occurs mainly in a limited time window post vaccination.

The main caveat of our study was the small sample size of both the WT and B.1.351 variants. These small samples sizes are a product of: the dramatic increase in frequency of the B.1.1.7 variant, first detected in Israel in mid-December 2020, and reaching an overall frequency of ~90% or higher during the period of this study (Fig. 1a); and the low frequency of the B.1.351 variant in Israel at the time of writing^16^. In fact, in our latest samples obtained in late February and early March 2021, we noted fixation of the B.1.1.7 variant, but this interpretation requires caution as our sample size was low (Fig. 1a). Furthermore, caution is required from overinterpreting the McNemar ORs obtained, for two reasons: statistically, they do not necessarily represent the OR of breakthrough; and the absolute numbers we found, in particular for B.1.351 infections, are very small.

Our study design was not intended to deduce vaccine effectiveness against either variant, as we observe VOCs conditioned on infection. In other words, our focus is only on infected individuals; we ignore non-infected individuals, and do not measure absolute infection rates in the vaccinated or control population. Thus, we can only cautiously speculate on vaccine effectiveness against the B.1.1.7 and B.1.351 strains. Previous real-world work has shown a very high effectiveness of the BNT162b2 vaccine starting a week after the second dose in a large-scale study performed in Israel among CHS patients^1^. During the period of that study, B.1.1.7 rose to a high frequency in Israel, suggesting that the high vaccine effectiveness observed in the study included high effectiveness against this strain as well. However, our current study may suggest a lower protection against B.1.1.7 in the first weeks after the first vaccine dose. As some countries opt to increase the gap between the first and the second BNT162b2 vaccine from the recommended 3 weeks to a longer period^21^, it is important to carefully assess whether this delay impacts vaccine effectiveness against the B.1.1.7 strain among individuals who received only the first dose. In our data we do not observe increased breakthrough of either the B.1.1.7 or the B.1.351 strain 2 weeks after the second dose, yet we note our data are relatively limited in this period (76 cases, Table 1). Our results are overall aligned with recent results that have shown slightly reduced vaccine effectiveness against the B.1.1.7 and B.1.351 variants as compared to WT, 14 days after the second dose^22^.

We conclude by discussing mechanistic explanations for why we see increased breakthrough rates at very particular and different time windows following vaccination for B.1.1.7 and B.1.351. From a biological point of view, the breakthrough cases observed in this study might be due to immune evasion, mediated by particular alterations present in these strains^9,23–27^. Alternatively, it has been previously reported that B.1.1.7 is associated with lower Ct values, corresponding to higher VLs^3^, which may be sufficient to overcome the less potent immune response elicited by the vaccine before its augmentation by a second dose. We note that in this study we did not observe higher VL in B.1.1.7 infections as compared to other variants (see also ref. ^28^), yet we did note higher VL in B.1.351 infections, while noting lower VL in vaccinees as compared to unvaccinated individuals (Supplementary Fig. 6)^29,30^. We stress that these findings are preliminary and may be affected by various behavioral biases. In particular, vaccinees in Israel were exempt from quarantine and testing following exposure to a positive patient, and this may affect when and how they chose to be tested. Moreover, this created a bias in their symptomatic status—most vaccinees were likely tested only when they were symptomatic. For this reason, we refrain from reporting the rate of symptoms in our cohort.

We were reassured to observe the low frequency of B.1.351 across time (Fig. 1a)^16^. Of note, both B.1.1.7 and B.1351 were first detected in Israel in late December, at the time vaccination commenced. Our sampling began during a peak of epidemic growth, during increasing rates of vaccination, into a phase of epidemic contraction (Fig. 1 and Supplementary Fig. 4). Owing to these complex dynamics, we can only speculate that selection does not strongly favor the B.1.351 variant in the particular conditions in Israel, despite the increased rate of vaccination. This may be due to its limited ability to evade vaccine-elicited immunity, mainly during days 7–13 after the second dose. Alternatively, from an evolutionary point of view, it is possible that immune evasion alterations incur a fitness cost in the form of reduced transmissibility, especially as compared to the highly transmissible B.1.1.7 (ref. ^3^). More research is required to further understand the evolutionary pressures operating on VOCs, in Israel, and around the world. At the time of revision of this paper, May 2021, we note that case counts in Israel have dramatically dropped to around 35 cases per day in a population of ~9 million (ref. ^31^), suggesting that while vaccine breakthrough infections at particular windows of time may be more frequent with the VOCs B.1.1.7 and B.1.351, mass vaccination with two doses controls and contains their spread.

## Methods

### Ethics statement

The study was approved by the CHS institutional review board (no. 0016-21-COM2) and was exempt from the requirement for informed consent. The study was further approved by the Tel Aviv University ethics committee (0002706-1).

### Sample matching

Data for this study were obtained from CHS’s data repositories (Supplementary Fig. 1). The study population consisted of individuals who tested positive for SARS-CoV-2 by PCR with reverse transcription from six major CHS testing laboratories located throughout Israel. Individuals with a positive PCR test were then classified into one of two groups: controls who were not vaccinated before the positive PCR result; and cases that were vaccinated at least 14 days before the PCR result. Cases were further divided into two additional subgroups: individuals who had a positive PCR test that was performed between 14 days after the first dose and 6 days after the second dose were denoted as the dose1 group, and individuals who had a positive PCR test that was performed at least 7 days after the second vaccine dose were denoted as the dose2 group. Next, each case was matched to a control using six parameters: date of sampling for PCR (±3 days), sex, age (±10 years), municipality of residence, geographical district of residence and sector. If two or more controls were available, one was chosen at random. In preliminary analyses, we noted that matching often failed for dose2 samples due to their small sample size, as well as due to increasing proportions of vaccinated individuals in older age categories across time, in line with the vaccine rollout policy in Israel. To increase dose2 matching, we enforced matching on the date of PCR sampling, but allowed for four out of five matches in the remaining parameters, while prioritizing municipality, then sector, then age, and then the additional criteria. We found that the failed parameter match was most often age, sex or municipality. We note that ten control samples served as controls for both a dose1 and a dose2 sample. Table 1 summarizes statistics on the parameters used for matching and other parameters for the various groups of our sample. We also note that some vaccinees (7.7%, Table 1) received the second vaccine dose more than 28 days after the first vaccine, yet were still annotated as described above.

### Obtaining RNA samples and sequencing

Following matching, RNA from cases and controls was obtained from the main testing laboratories of CHS, with one major limitation: only samples with Ct values of 33 or lower were collected. Ct values were averaged over all genes tested (per laboratory). The dates of the samples ranged from 23 January 2021 to 7 March 2021 (Supplementary Fig. 4). Full-genome sequencing of SARS-CoV-2 was performed based on the ARTIC protocol with a V3 primer set (https://artic.network/ncov-2019), with slight modifications detailed below. Briefly, reverse transcription and multiplex PCR was performed in two amplicon pools, and NEBNext Multiplex Oligos for Illumina were ligated to allow for sequencing. All samples were run on an Illumina MiSeq using 250-cycle V2 kits at either the Technion Genome Center (Israel) or at the Genomics Research Unit at Tel Aviv University (Israel). We and others have previously noted amplicon dropout of amplicons 74 and 76 (ref. ^32^), both of which cover the spike gene, and in particular some of the lineage-defining alterations of B.1.1.7 and B.1.351 (such as E484K and N501Y). To increase the sequencing yield of these amplicons, we doubled the primer concentrations of both amplicons in our primer pool and lowered the annealing–extension temperature to 63 °C.

### Bioinformatic analysis and lineage assignment

Sequencing reads were trimmed using pTrimmer v1.3.1, a multiplexing primer trimming tool^33^, and then aligned to the reference genome of SARS-CoV-2 (GenBank ID MN908947) using our AccuNGS V1 pipeline^34^ that is based on BLAST^35^, using a particular stringent e-value of 10^−30^. We then set out to determine the consensus sequence of each sample. Typically studies report a majority-rule consensus sequence; that is, the consensus base at each position in the genome is the base that most reads (>50%) support. However, the biological meaning of variable positions where more than one base is observed is complex, especially if such positions are abundant: they may indicate within-host variation, they may indicate multiple genotype infection, they may indicate sample contamination, and they may indicate sequencing errors. To overcome these limitations, we constructed two consensus sequences for each sample, one based on majority rule, and a more strict consensus sequence where we required at least 80% of reads to support a given base. Bases with lower support were assigned an N (ambiguous base). We also noted some regions with fluctuating ambiguity: if a continuous subsequence of length 20 or lower was flanked on both ends by ambiguous bases, we masked out this entire subsequence by assigning it with N, under the assumption it represents unreliable sequencing. In both types of consensus sequencing assignments, we required sequencing coverage of at least ten reads. Finally, we used the Pangolin v2.2.2 software (https://github.com/cov-lineages/pangolin) to assign lineages for each consensus sequence using the Pango nomenclature (pangoLEARN 2021-02-06)^36^, which requires that at least 50% of bases sequenced are unambiguous. After verifying the type of lineages we obtained, we labeled all consensus sequences as either B.1.1.7, B.1.351 or WT. Samples for which Pangolin labels of the strict and majority-rule consensuses did not coincide were discarded. Thirty-two pairs in which one sample did not undergo successful sequencing were discarded from the paired analyses (but see Supplementary Fig. 3). The unpaired successful samples were, however, included in the variant frequencies across time analysis (Fig. 1a).

Following classification by Pangolin, we noted that one dose1 control sequence, originally classified as WT (B.1.235), was located within the B.1.351 clade on the phylogenetic tree. Its pair was classified as B.1.1.7, and we included this pair in our extreme scenarios analysis (Supplementary Fig. 3). This is in line with recent concerns regarding misclassifications of Pangolin^37^, and led us to manually verify the phylogenetic location of all sequences in our study.

R v4.0.4, Python v3.7.4, pandas v0.24.2 (ref. ^38^), Matplotlib v3.2.1 (ref. ^39^), Seaborn v0.10.1 and ggplot2 (ref. ^40^) were used during the data analysis and visualization.

### Statistical analysis

For all primary analyses, a one-sided paired (exact) McNemar’s test was used to compare breakthrough of a variant in partially or fully vaccinated individuals. For the analysis of B.1.351, all other variants were defined as the reference group, while for the B.1.1.7 analysis, we excluded any paired observation that included B.1.3.5 (assuming ordinality of breakthrough), while any other variant was defined as the reference. A conditional logistic regression was used as a sensitivity analysis to include age as a possible confounder in case that matching was not sufficient, under the assumption that it was sometimes only partially mediated through matching. The regression was performed only on the dose1 B.1.1.7 analysis, as not enough data were available in other categories. All analyses were conducted with R software version 4.03 and the survival and exact2x2 packages.

### Phylogenetic analysis

All Israeli sequences available on GISAID (https://www.gisaid.org/) from August onwards were downloaded, focusing on high-quality sequences with 10% or less ambiguous sites. Of these sequences, owing to computational limitations, we sampled the most distant 100 WT sequences and 50 B.1.1.7 sequences, and included all available B.1.351 sequences (Supplementary Table 2). The reference genome sequence (MN908947.3) was added on as well, and these sequences were combined with sequences from this study that contained at most 10% ambiguous sites. Alignment was performed using Mafft v7.300b (ref. ^41^) with default parameters. Next, a maximum-likelihood phylogeny was reconstructed using PhyML v3.0_360-500M (ref. ^42^) with default parameters as well, and the tree was rooted using the MN908947.3 sequence from the original outbreak first detected in Wuhan. The ggtree v2.5.1 package was used to visualize the phylogenetic trees.

### Reporting Summary

Further information on research design is available in the Nature Research Reporting Summary linked to this article.

## Online content

Any methods, additional references, Nature Research reporting summaries, source data, extended data, supplementary information, acknowledgements, peer review information; details of author contributions and competing interests; and statements of data and code availability are available at 10.1038/s41591-021-01413-7.

## Supplementary information

Supplementary Information Supplementary Figs. 1–6.

Reporting Summary

Supplementary Table 1 A list of all sequence accession numbers used in this study and their metadata.

Supplementary Table 2 A list of all additional publicly available Israeli sequences from GISAID used in the study.

## Data Availability

All sequences were uploaded to GISAID and the accession numbers are stated in Supplementary Table 1. The raw sequencing reads were deposited in the National Center for Biotechnology Information Sequence Read Archive database under BioProject accession number PRJNA728463.

## Code availability

Code used to generate consensus sequences as described in the section entitled Bioinformatic analysis and lineage assignment is available at https://github.com/SternLabTAU/COVID19-VACC.
