# Supplementary material for: Evidence for increased breakthrough rates of SARS-CoV-2 variants of concern in BNT162b2-mRNA-vaccinated individuals
Source: Nat Med. 2021 Jun 14;27(8):1379–84. doi: 10.1038/s41591-021-01413-7 (PMC8363499; doi:10.1038/s41591-021-01413-7)
Supplement: Supplementary file 1 — Supplementary Figs. 1–6. [file 41591_2021_1413_MOESM1_ESM.pdf]

nature portfolio

https://doi.org/10.1038/s41591-021-01413-7

Supplementary information

Evidence for increased breakthrough rates of SARS-CoV-2 variants of concern in BNT162b2-mRNA-vaccinated individuals

In the format provided by the authors and unedited

Supplementary figures for "Evidence for increased breakthrough rates of SARS-CoV-2 variants of concern in BNT162b2 mRNA vaccinated individuals"

Talia Kustin*[1,2], Noam Harel*[1,2], Uriah Finkel[3], Shay Perchik[3], Sheri Harari[1,2], Maayan Tahor[1], Itamar Caspi[1], Rachel Levy[1], Michael Leshchinsky[3], Shifra Ken Dror[4], Galit Bergerzon[4], Hala Gadban[4], Faten Gadban[4], Eti Eliassian[5], Orit Shimron[5], Loulou Saleh[6], Haim Ben-Zvi[6], Elena Keren Taraday[7], Doron Amichay[7,8], Anat Ben-Dor[7], Dana Sagas[9], Merav Strauss[9], Yonat Shemer Avni[10,11], Amit Huppert[12,13], Eldad Kepten[3], Ran D. Balicer[3], Doron Nezer[14*], Shay Ben-Shachar[3,13]*†, Adi Stern[1,2]†

* These authors contributed equally
† Corresponding authors: shayb@clalit.org.il, sternadi@tauex.tau.ac.il

1. The Shmunis School of Biomedicine and Cancer Research, George S. Wise Faculty of Life Sciences, Tel Aviv University, Tel Aviv, Israel.
2. Edmond J. Safra Center for Bioinformatics, Tel Aviv University, Tel Aviv, Israel
3. Clalit Research Institute, Innovation Division, Clalit Health Services, Ramat Gan, Israel
4. Clalit Health Services, Central Laboratories, Haifa and Western Galilee, Israel
5. Progenin Laboratories, Jerusalem District, Clalit Health Services, Israel
6. Microbiology lab, Rabin Medical Center, Beilinson Hospital, Petach Tikva, Israel
7. Central Laboratory, Clalit Health Services, Tel Aviv, Israel
8. Department of Clinical Biochemistry and Pharmacology, Faculty of Health Sciences, Ben-Gurion University, Beersheba, Israel
9. Microbiology Laboratory, Emek Medical Center, Afula, Israel
10. Laboratory of Clinical Virology, Soroka University Medical Center. Beersheba, Israel
11. Faculty of Health Sciences, Ben Gurion University of the Negev, Beersheba, Israel
12. The Bio-statistical and Bio-mathematical Unit, The Gertner Institute for Epidemiology and Health Policy Research, Chaim Sheba Medical Center, Tel Hashomer, Israel
13. The Sackler Faculty of Medicine, Tel-Aviv University, Tel-Aviv, Israel
14. Clalit Health Services, Tel Aviv, Israel

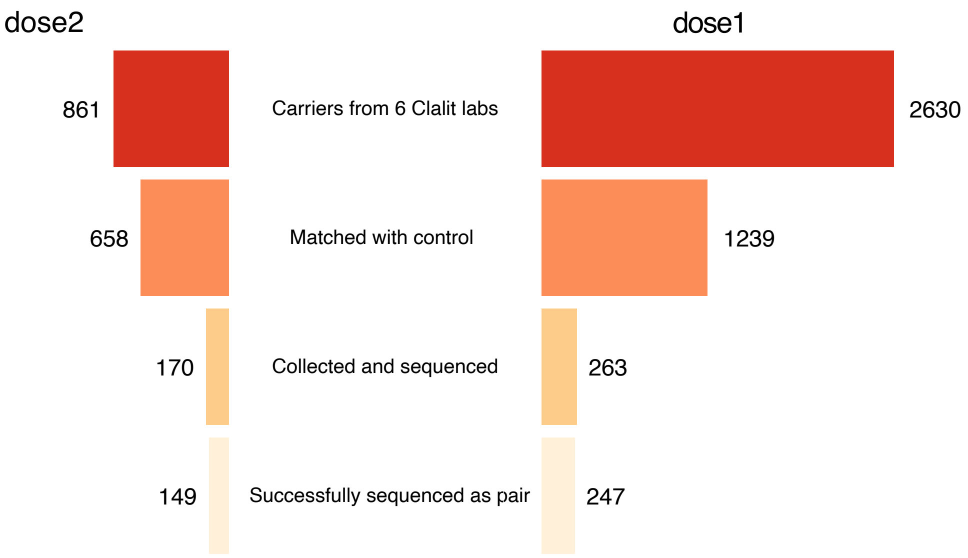

Fig. S1. Number of vaccinated carriers, from initial identification to successful sequencing. Matching with a control often failed due to two main reasons: (a) increasing proportions of the population were vaccinated, leaving smaller numbers of unvaccinated controls, (b) our strict requirement for matching of geographic location.

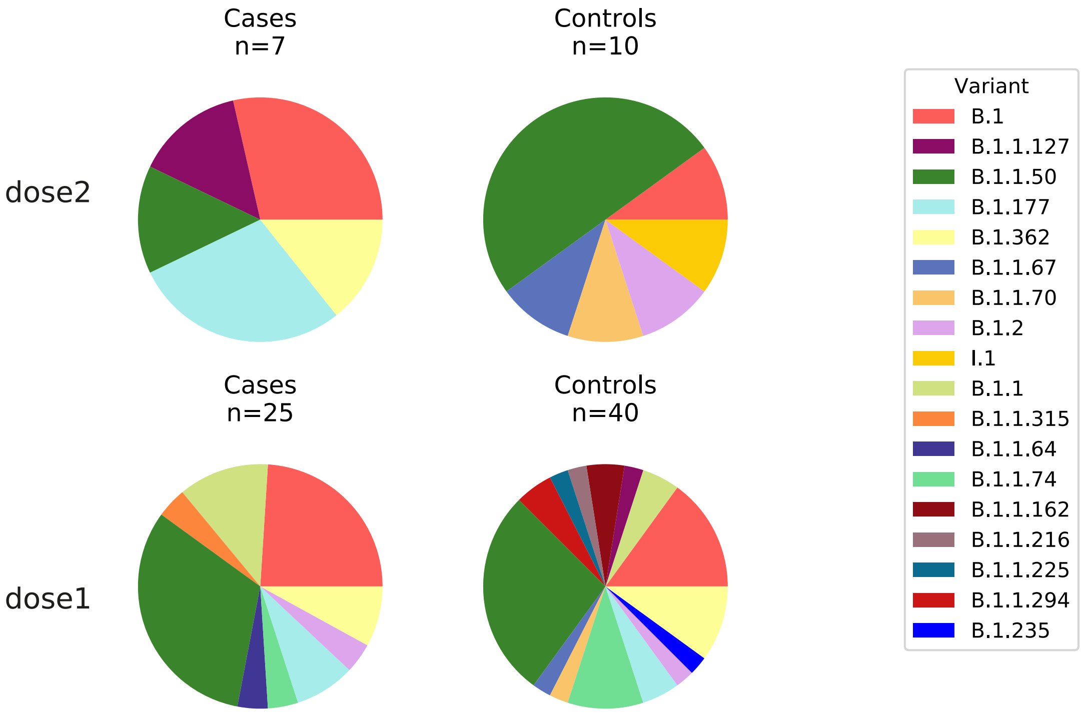

Fig. S2. WT variants (lineages) found in this study, partitioned into the four groups studied herein. Lineages were inferred based on strict consensus sequences (Methods). During revision of this paper, I.1 was found to be obsolete and was inferred to be B.1.1.50 using Pangolin version v.2.4.2, lineages version 2021-05-19.

(A)

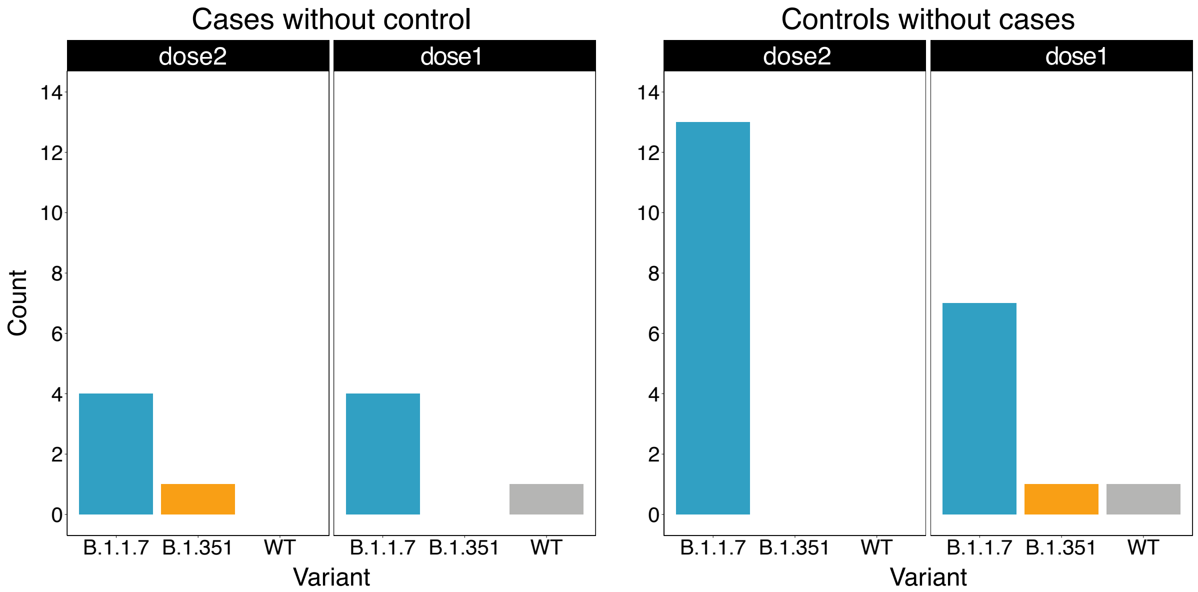

(B)

Scenario that most strengthens our results:

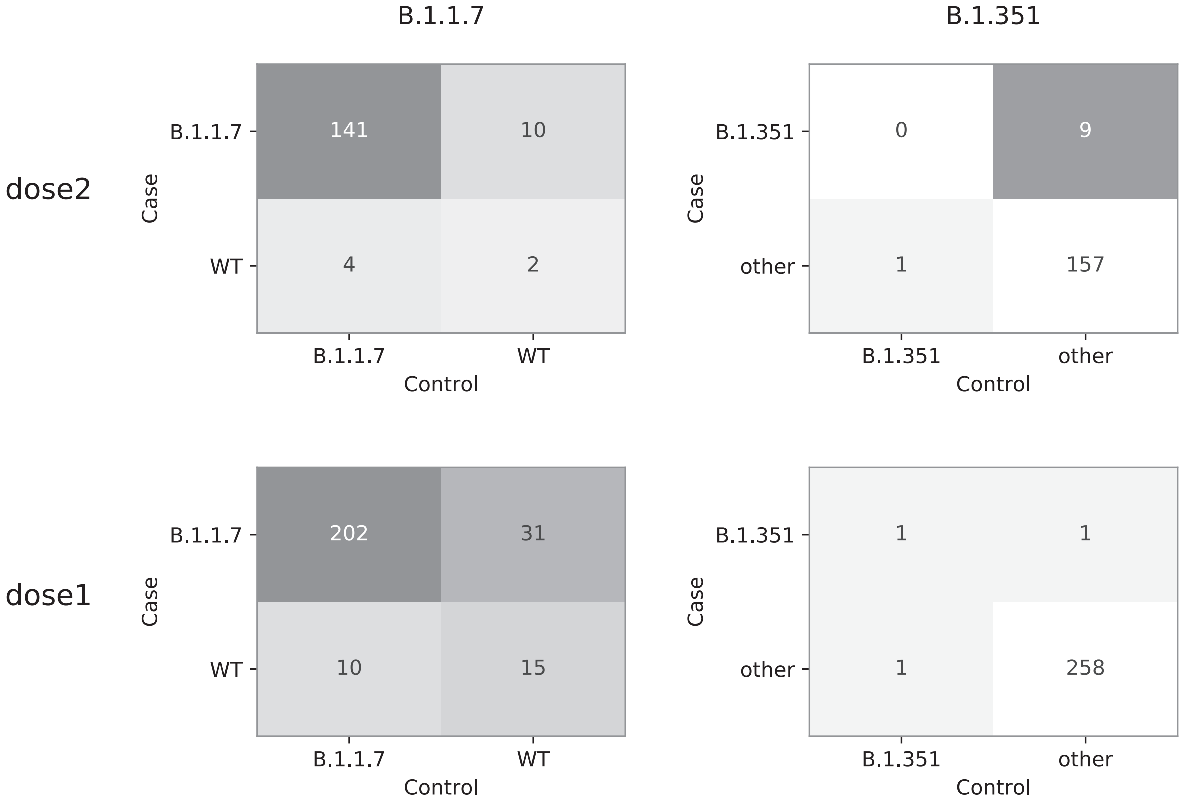

Scenario that most disrupts our results:

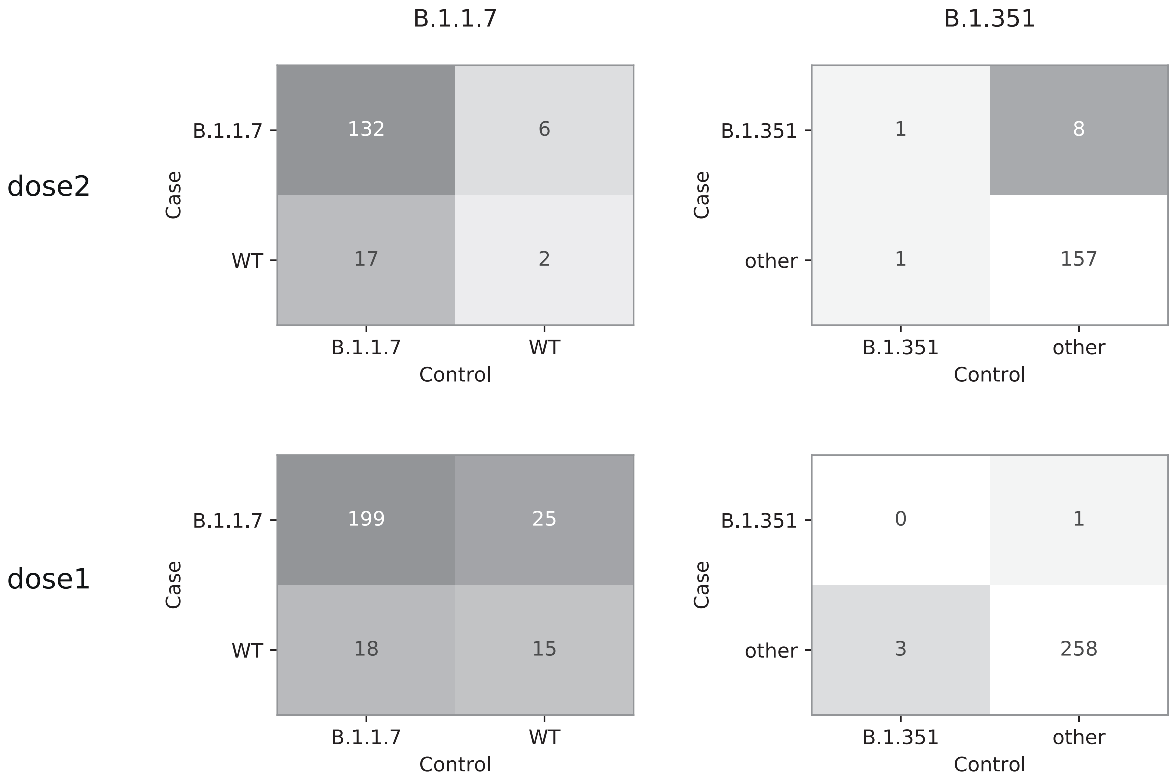

Figure S3. Sequences whose match (case or control) were not sequenced, or whose pangolin classification was questionable. (A) Counts of sequences based on their category (case/control and FE/PE) and variant assignment. (B) We theoretically assign the sequences into the two most extreme scenarios: a scenario that most strengthens our original results as reported in the main text (upper panel), versus a scenario that is most disruptive to the original results (lower panel). Under both scenarios, our results on B.1.351 remain unchanged, with the p-value of the Mcenmar test dropping to 0.01. Results on B.1.1.7 remain qualitatively similar. Under the strengthening scenario the McNemar p-value drops to $7x10^{-4}$, but under the disruptive scenario the McNemar p-value rises to 0.15. We speculate that the true scenario is most likely somewhere in between both extremes.

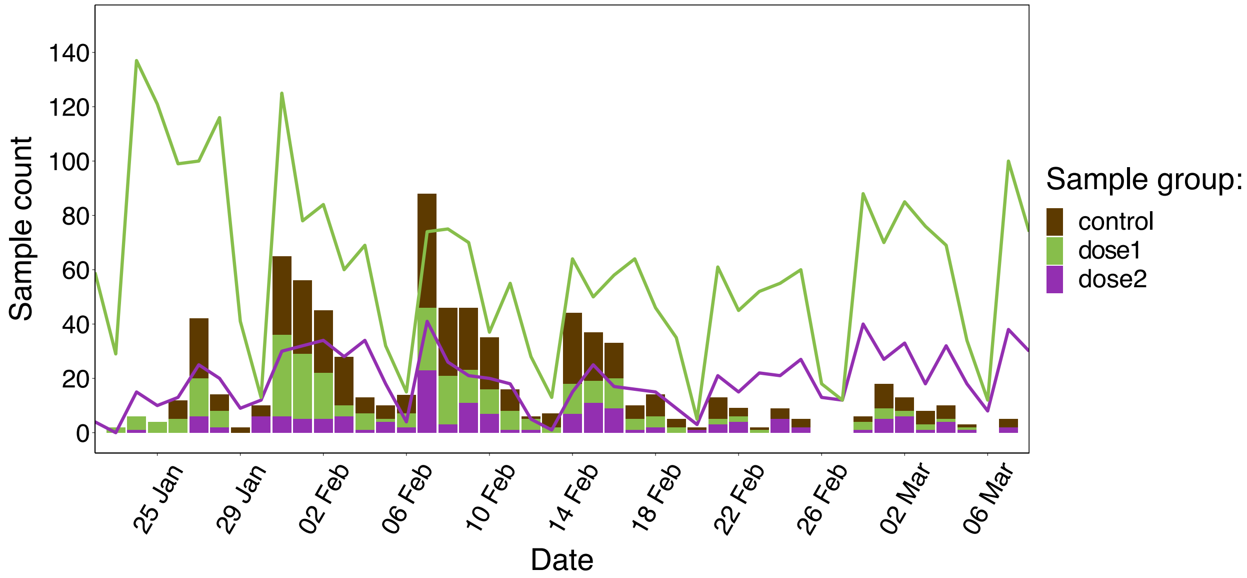

Fig. S4. Numbers of daily vaccinated carriers. Data are separated by effectiveness (full effectiveness and partial effectiveness, as defined in the main text) from the six major CHS testing labs located throughout Israel. The bars represent the daily numbers of vaccinees and controls that were successfully sequenced.

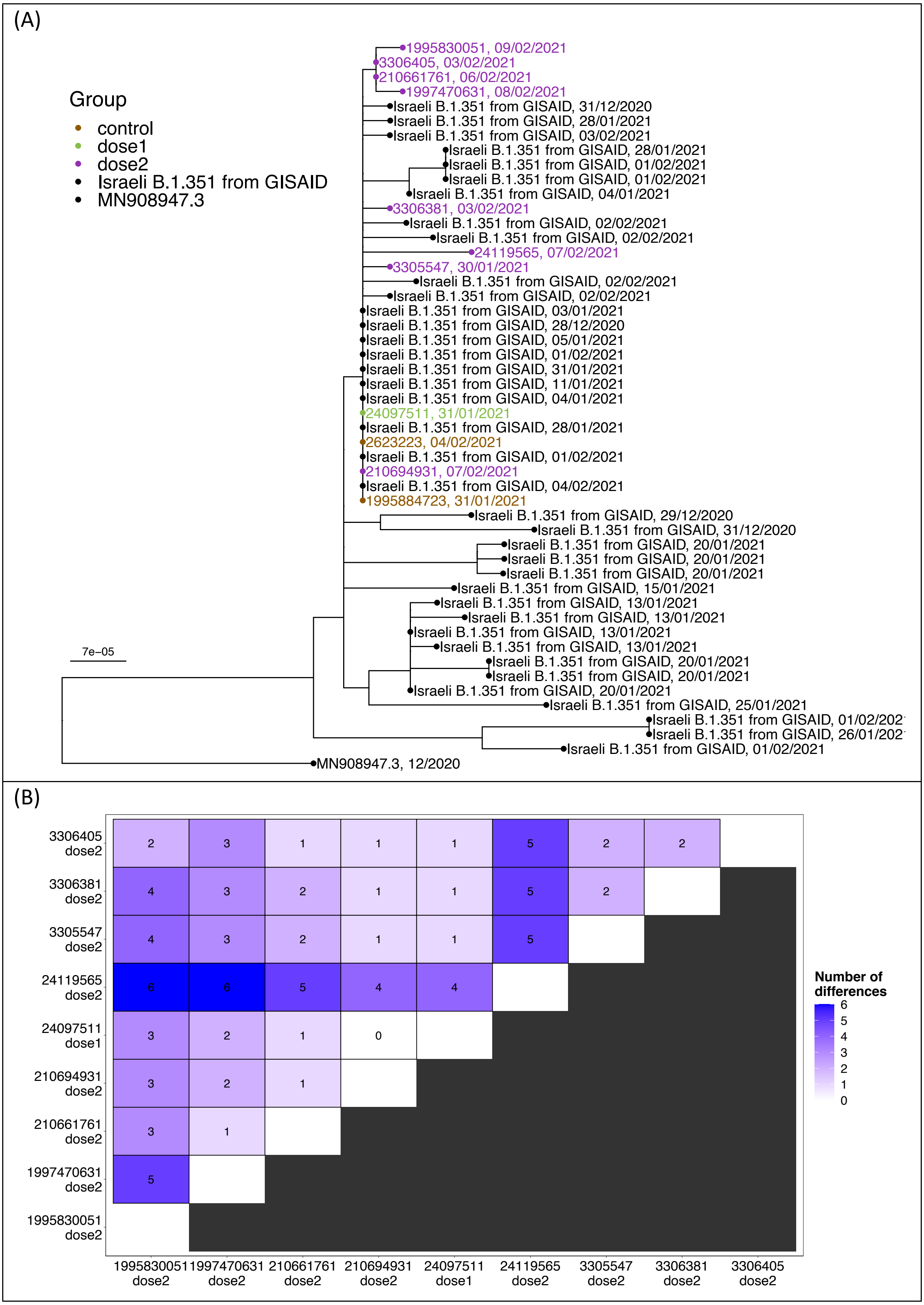
(A)
Group
control
dose1
dose2
Israeli B.1.351 from GISAID
MN908947.3
1995830051, 09/02/2021
3306405, 03/02/2021
210661761, 06/02/2021
1997470631, 08/02/2021
Israeli B.1.351 from GISAID, 31/12/2020
Israeli B.1.351 from GISAID, 28/01/2021
Israeli B.1.351 from GISAID, 03/02/2021
Israeli B.1.351 from GISAID, 28/01/2021
Israeli B.1.351 from GISAID, 01/02/2021
Israeli B.1.351 from GISAID, 01/02/2021
Israeli B.1.351 from GISAID, 04/01/2021
3306381, 03/02/2021
Israeli B.1.351 from GISAID, 02/02/2021
Israeli B.1.351 from GISAID, 02/02/2021
24119565, 07/02/2021
3305547, 30/01/2021
Israeli B.1.351 from GISAID, 02/02/2021
Israeli B.1.351 from GISAID, 02/02/2021
Israeli B.1.351 from GISAID, 03/01/2021
Israeli B.1.351 from GISAID, 28/12/2020
Israeli B.1.351 from GISAID, 05/01/2021
Israeli B.1.351 from GISAID, 01/02/2021
Israeli B.1.351 from GISAID, 31/01/2021
Israeli B.1.351 from GISAID, 11/01/2021
Israeli B.1.351 from GISAID, 04/01/2021
24097511, 31/01/2021
Israeli B.1.351 from GISAID, 28/01/2021
2623223, 04/02/2021
Israeli B.1.351 from GISAID, 01/02/2021
210694931, 07/02/2021
Israeli B.1.351 from GISAID, 04/02/2021
1995884723, 31/01/2021
Israeli B.1.351 from GISAID, 29/12/2020
Israeli B.1.351 from GISAID, 31/12/2020
Israeli B.1.351 from GISAID, 20/01/2021
Israeli B.1.351 from GISAID, 20/01/2021
Israeli B.1.351 from GISAID, 20/01/2021
Israeli B.1.351 from GISAID, 15/01/2021
Israeli B.1.351 from GISAID, 13/01/2021
Israeli B.1.351 from GISAID, 13/01/2021
Israeli B.1.351 from GISAID, 13/01/2021
Israeli B.1.351 from GISAID, 13/01/2021
Israeli B.1.351 from GISAID, 20/01/2021
Israeli B.1.351 from GISAID, 20/01/2021
Israeli B.1.351 from GISAID, 20/01/2021
Israeli B.1.351 from GISAID, 25/01/2021
Israeli B.1.351 from GISAID, 01/02/202
Israeli B.1.351 from GISAID, 26/01/202
Israeli B.1.351 from GISAID, 01/02/2021
7e−05
MN908947.3, 12/2020
(B)
3306405 dose2: 2 3 1 1 1 5 2 2
3306381 dose2: 4 3 2 1 1 5 2
3305547 dose2: 4 3 2 1 1 5
24119565 dose2: 6 6 5 4 4
24097511 dose1: 3 2 1 0
210694931 dose2: 3 2 1
210661761 dose2: 3 1
1997470631 dose2: 5
1995830051 dose2
1995830051 dose2
1997470631 dose2
210661761 dose2
210694931 dose2
24097511 dose1
24119565 dose2
3305547 dose2
3306381 dose2
3306405 dose2
Number of differences
6
5
4
3
2
1
0

Fig S5. Zoom-in on the B.1.351 samples. (A) A maximum-likelihood phylogenetic tree of all B.1.351 samples identified in the study, as well as sequences of B.1.351 from Israel publicly available on GISAID. The tree was rooted using the MN908947.3 reference sequence from Wuhan, and internal branches whose length was less than $3x10^{-5}$ (corresponding to less than one substitution along the genome) were collapsed . Each sequence was coloured based on the sample group, and sampling dates were added on as well. (B) A heatmap of the number of pairwise substitutions between all pairs of B.1.351 dose2/dose1 sequences found in the study. Substitutions included point mutations and indels, and were all verified manually. Ambiguous positions corresponding to unreliable sequencing (Methods) were excluded from the analysis. We note that sequencing errors have been noted in some SARS-CoV-2 samples, most often in samples with high Ct (typically higher than 25) [1, 2]. Six of the B.1.351 samples presented here had Ct values lower than 25 and even lower than 20 (Fig. S7), suggesting that the differences reported here are reliable.

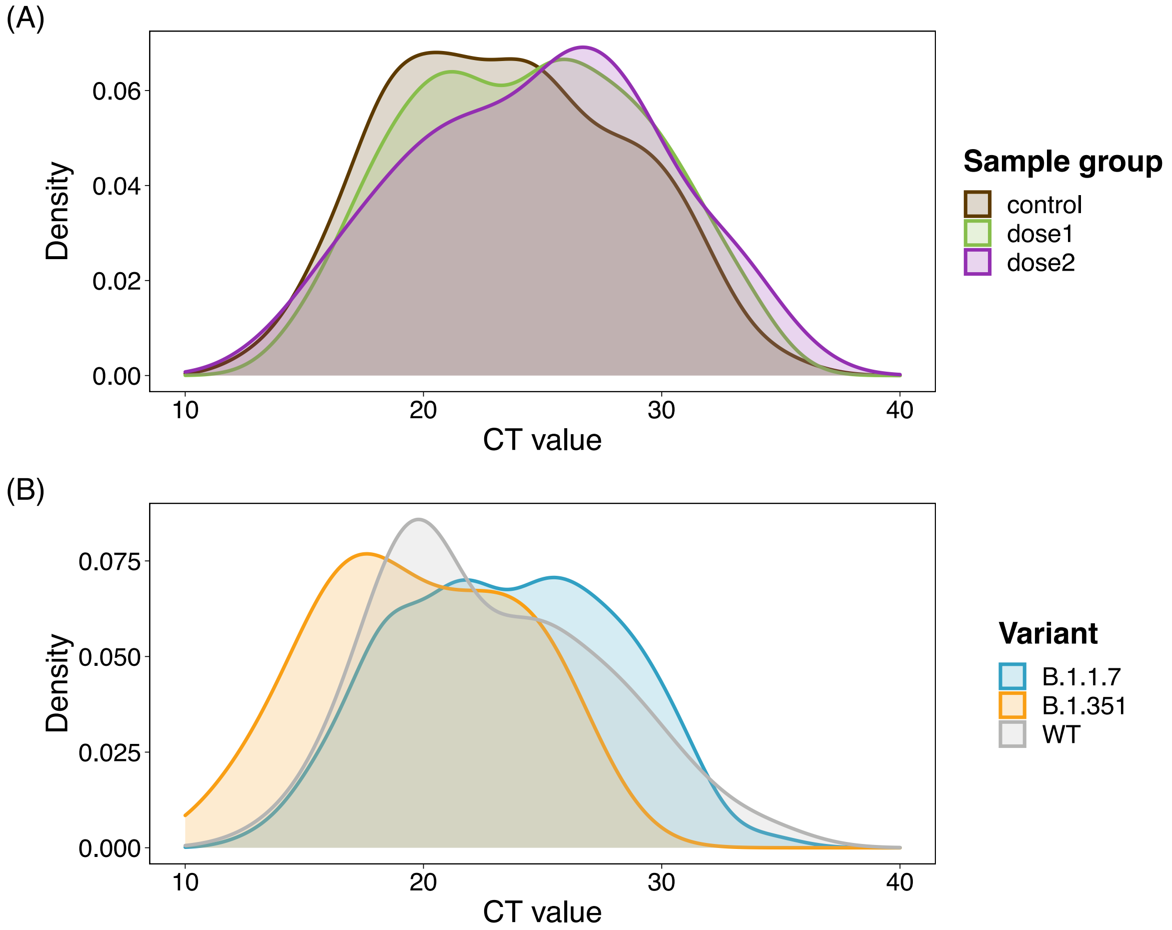

Fig S6. Density plots of Ct values found in the samples collected in this study. (A) Ct values distributed by groups in the study (Control, dose1 and dose2). Values are shown only if both case and control values were available (but not necessarily all were sequenced, see main text). The data for this plot contains Ct values of 223 dose1 samples and 144 dose2 samples, and their paired controls. (B). Ct values distributed by variant. The data for this plot contains 75 WT samples, 547 B.1.1.7 samples and 11 B.1.351 samples. For (A) and (B), we note that for some samples the exact Ct value was missing (only its range was reported), and hence the smaller number of samples as compared to the main text. Moreover, only samples with Ct values of 33 or lower were collected (Methods), causing our data to be consistently biased towards lower values, across all categories of groups.

References

1. Lythgoe, K.A., et al., *SARS-CoV-2 within-host diversity and transmission.* Science, 2021. **372**(6539).
2. Tonkin-Hill, G., et al., *Patterns of within-host genetic diversity in SARS-CoV-2.* bioRxiv, 2020: p. 2020.12.23.424229.
