# Supplementary material for: Evidence for increased breakthrough rates of SARS-CoV-2 variants of concern in BNT162b2-mRNA-vaccinated individuals
Source: Nat Med. 2021 Jun 14;27(8):1379–84. doi: 10.1038/s41591-021-01413-7 (PMC8363499; doi:10.1038/s41591-021-01413-7)
Supplement: Supplementary file 2 — Reporting Summary [file 41591_2021_1413_MOESM2_ESM.pdf]

nature research

Corresponding author(s): Shay Ben-Shachar, Adi Stern

Last updated by author(s): May 19, 2021

Reporting Summary

Nature Research wishes to improve the reproducibility of the work that we publish. This form provides structure for consistency and transparency in reporting. For further information on Nature Research policies, see our Editorial Policies and the Editorial Policy Checklist.

Statistics

For all statistical analyses, confirm that the following items are present in the figure legend, table legend, main text, or Methods section.

n/a	Confirmed	
☐	☒	The exact sample size (n) for each experimental group/condition, given as a discrete number and unit of measurement
☐	☒	A statement on whether measurements were taken from distinct samples or whether the same sample was measured repeatedly
☐	☒	The statistical test(s) used AND whether they are one- or two-sided<br>*Only common tests should be described solely by name; describe more complex techniques in the Methods section.*
☐	☒	A description of all covariates tested
☐	☒	A description of any assumptions or corrections, such as tests of normality and adjustment for multiple comparisons
☐	☒	A full description of the statistical parameters including central tendency (e.g. means) or other basic estimates (e.g. regression coefficient) AND variation (e.g. standard deviation) or associated estimates of uncertainty (e.g. confidence intervals)
☐	☒	For null hypothesis testing, the test statistic (e.g. F, t, r) with confidence intervals, effect sizes, degrees of freedom and P value noted<br>*Give P values as exact values whenever suitable.*
☒	☐	For Bayesian analysis, information on the choice of priors and Markov chain Monte Carlo settings
☐	☒	For hierarchical and complex designs, identification of the appropriate level for tests and full reporting of outcomes
☒	☐	Estimates of effect sizes (e.g. Cohen's d, Pearson's r), indicating how they were calculated

Our web collection on statistics for biologists contains articles on many of the points above.

Software and code

Policy information about availability of computer code

Data collection	None
Data analysis	pTrimmer v1.3.1, AccuNGS V1, pangolin 2.2.2, pangoLEARN 2021-02-06, R software version 4.03 and the survival and exact2x2 packages, Python v3.7.4, pandas v0.24.2, Matplotlib v3.2.1, Seaborn v0.10.1, ggplot2 v3.3.3, ggtree v2.5.1, MAFFT v7.300b, PhyML v3.0_360-500M, BLAST. All software used is cited.

For manuscripts utilizing custom algorithms or software that are central to the research but not yet described in published literature, software must be made available to editors and reviewers. We strongly encourage code deposition in a community repository (e.g. GitHub). See the Nature Research guidelines for submitting code & software for further information.

Data

Policy information about availability of data

All manuscripts must include a data availability statement. This statement should provide the following information, where applicable:

- Accession codes, unique identifiers, or web links for publicly available datasets
- A list of figures that have associated raw data
- A description of any restrictions on data availability

Data that support the findings of this study have been deposited in the relevant databases: a viral sequence per each patient sample was deposited in the GISAID database (https://www.gisaid.org) and accession numbers are stated in the study (Table S1). The raw sequencing reads were deposited in the NCBI Sequence Read Archive (SRA) database under BioProject accession number PRJNA728463 (https://www.ncbi.nlm.nih.gov/bioproject/PRJNA728463). The reference genome of SARS-CoV-2, ID MN908947, was downloaded from GenBank (https://www.ncbi.nlm.nih.gov/genbank/) and the list of all sequence accession numbers used in this study is available in table S2.

Field-specific reporting

Please select the one below that is the best fit for your research. If you are not sure, read the appropriate sections before making your selection.

☐ Life sciences ☐ Behavioural & social sciences ☒ Ecological, evolutionary & environmental sciences

For a reference copy of the document with all sections, see nature.com/documents/nr-reporting-summary-flat.pdf

Ecological, evolutionary & environmental sciences study design

All studies must disclose on these points even when the disclosure is negative.

Study description	SARS-CoV-2 samples were collected from infected individuals. Samples of vaccinees with the BNT162b2 mRNA vaccine were matched with samples from non-vaccinees. All samples were sequenced and the variant of the virus was determined.
Research sample	RNA extracted from nasopharynx swabs of SARS-CoV2 positive individuals was collected by six major Clalit Health Services testing labs located throughout Israel for the purpose of testing for COVID-19 (not related to this research). This study used leftovers of these samples. Samples were collected across the time interval of January 23, 2021 and March 07, 2021. The samples included both males and females of all ages. Our sample consisted of the vaccinated infected individuals that were sampled in one of the six laborotories of Clalit Health Services and that were successfully matched with an unvaccinated control (See Fig S1)
Sampling strategy	Each sample of a vaccinated individual was matched to a sample of a non-vaccinated individual based on the following parameters: date of sampling for PCR (+/- three days), sex, age (+/- 10 years), municipality of residence, geographical district of residence, and sector.<br>We performed power analysis in order to determine the desired sample size. Since the B.1.351 frequency in Israel prior to our research was estimated at 1%, our analysis resulted in a desired sample size of 800-1000 samples (400-500 matched pairs). We have reached close to 400 pairs and concluded our sampling mainly due to the decline in the number of cases in Israel and more specifically almost no infected vaccinees.
Data collection	Extracted RNA was collected and delivered by Clalit Health Services for sequencing in the Genomic centers at the Technion or at Tel Aviv University. Metadata of samples was provided by Clalit Health Services and included sampling date, age, sex and vaccination status. Data regarding the match of each sample of vaccinee according to the matching scheme was provided by Clalit Health Services.
Timing and spatial scale	Samples were collected starting on January 23, 2021 till March 07, 2021 from six major Clalit Health Services testing labs that are spread across Israel. Our sampling started at the start of the reaserch and our end date was when we reached around 800 samples as described above.
Data exclusions	Samples whose sequencing results were unsuccessful were excluded from the analysis. This led to the exclusion of pairs of sequences: if a control or case was excluded, their paired case or control was excluded, respectively. .
Reproducibility	There is no possibility to reproduce this type of cohort study, especially given the small numbers of infected vaccinated individuals.
Randomization	Allocation to groups was made based on vaccination status. Covariates were controlled to as much as possible using our matching scheme between vaccinees and non-vaccinees based on the following parameters: date of sampling for PCR (+/- three days), sex, age (+/- 10 years), municipality of residence, geographical district of residence, and sector.
Blinding	All samples were anonymized, and were assigned a random identifier by Clalit Health Services

Did the study involve field work? ☐ Yes ☒ No

Reporting for specific materials, systems and methods

We require information from authors about some types of materials, experimental systems and methods used in many studies. Here, indicate whether each material, system or method listed is relevant to your study. If you are not sure if a list item applies to your research, read the appropriate section before selecting a response.

Materials & experimental systems

n/a	Involved in the study	
☒	☐	Antibodies
☒	☐	Eukaryotic cell lines
☒	☐	Palaeontology and archaeology
☒	☐	Animals and other organisms
☐	☒	Human research participants
☒	☐	Clinical data
☒	☐	Dual use research of concern

Methods

n/a	Involved in the study	
☒	☐	ChIP-seq
☒	☐	Flow cytometry
☒	☐	MRI-based neuroimaging

Human research participants

Policy information about studies involving human research participants

Population characteristics	SARS-CoV-2 samples were collected from infected individuals. Samples of vaccinees with the BNT162b2 mRNA vaccine were matched with samples from non-vaccinees. Each sample of a vaccinated individual was matched to a sample of a non-vaccinated individual by the following parameters: date of sampling for PCR (+/- three days), sex, age (+/- 10 years), municipality of residence, geographical district of residence, and sector. Aggregated population data is available in Table 1.
Recruitment	No direct recruitment was performed. This was a retrospective study, that relied on leftover samples from routine testing. Biases may include the subjective inclination of individuals to go and get tested and the fact we discarded samples with high CTs (low viral load) and their pairs due to inability to sequence those samples. These biases are described in the main text.
Ethics oversight	The study was approved by the Clalit Health Services institutional review board (IRB #0016-21-COM2) and was exempt from the requirement for informed consent. The study was further approved by the Tel-Aviv University ethics committee (0002706-1).

Note that full information on the approval of the study protocol must also be provided in the manuscript.
